# CD146 is a potential marker for the diagnosis of malignancy in cervical and endometrial cancer

**DOI:** 10.3892/ol.2013.1147

**Published:** 2013-01-22

**Authors:** HAOFENG ZHANG, JUN ZHANG, ZHAOQING WANG, DI LU, JING FENG, DONGLING YANG, XIUQIN CHEN, XIYUN YAN

**Affiliations:** 1 Department of Obstetrics and Gynecology, Capital Medical University Affiliated Beijing Anzhen Hospital, Institute of Beijing Heart, Lung and Blood Vessel Diseases, Chaoyang, Beijing 100029;; 2 Protein and Peptide Pharmaceutical Laboratory, Chinese Academy of Sciences-University of Tokyo Joint Laboratory of Structural Virology and Immunology, Institute of Biophysics, Chinese Academy of Sciences, Chaoyang, Beijing 100101;; 3 Department of Obstetrics and Gynecology, The Fifth Affiliated Hospital of Zhengzhou University, Erqi, Zhengzhou 450000, P.R. China

**Keywords:** CD146, marker, diagnostic testing, anti-CD146 mAb AA4, cervical cancer, endometrial cancer

## Abstract

Cluster of differentiation 146 (CD146) is an endothelial cell adhesion molecule which is overexpressed in various types of malignant cancer, including ovarian cancer. However, whether CD146 is overexpressed in another two types of gynecological cancer, cervical cancer and endometrial cancer, remains unclear. In the present study, we showed that CD146 expression levels were higher in cells from cervical cancer and endometrial cancer compared with their corresponding normal tissues, using anti-CD146 mouse antibody AA4 (mAb AA4) and that mAb AA4 exhibited a high performance for specificity, sensitivity and positive predictive value in the detection of these two types of cancer. CD146 expression was positively and significantly correlated with the pathological subtype of cervical cancer and with the histological grade and depth of myometrial invasion in endometrial cancer. In addition, we confirmed that CD146 is present in the majority of blood vessels in cervical and endometrial cancer, suggesting that CD146 may be actively implicated in the metastasis of cervical and endometrial cancer via the vascular system. Thus, this study provides insights for further development of CD146 mAb in the detection of gynecological malignant cancer types and implies that a combined treatment strategy of anti-CD146 immunotherapy with other traditional chemo- or radiotherapy treatments may be a promising approach against cervical and endometrial cancer.

## Introduction

Cluster of differentiation 146 (CD146) is a cell adhesion molecule (CAM) which belongs to the immunoglobulin superfamily (IgSF) (1). Human CD146 has previously been designated several synonyms, including MUC18 (1,2), A32 antigen (3,4), S-Endo-1 (5), melanoma CAM (MCAM or Mel-CAM) (4,6,7), metastasis CAM (MET-CAM) (8) and hemopoietic CAM (HEMCAM) (9). The avian homolog of CD146 has been named gicerin (10). CD146 was originally identified as a marker for melanoma (MCAM), due to its overexpression in metastatic lesions and advanced primary tumors, yet not in benign lesions (1,3,4). Increasing amounts of evidence have demonstrated that CD146 is overexpressed in a variety of carcinomas, in addition to melanomas (11–14). As a result of this characteristic, CD146 has attracted attention and is considered to be a potential marker for tumor diagnosis, prognosis and treatment. The majority of studies support the theory that CD146 promotes tumor growth, angiogenesis and metastasis (15), therefore, CD146 is a promising target for tumor therapy (16,17).

CD146 is a 113-kDa integral membrane glycoprotein, whose sequence of amino acids consists of a signal peptide, an extracellular fragment structure of five immunoglobulin-like domains (V-V-C2-C2-C2), a transmembrane region and a short cytoplasmic tail (2,18). The Protein and Peptide Pharmaceutical Laboratory, Chinese Academy of Sciences-University of Tokyo, Tokyo, raised an array of mouse antibodies (mAbs) against CD146, among which AA4 recognizes the epitope located at the second IgC2 domain (19). AA4 is able to specifically detect CD146 in tumor specimens from various organs, including breast tumors (14). CD146 overexpression has been revealed in ovarian cancer and may be a marker for poor prognosis in ovarian cancer patients (11). Thus, CD146 Abs may be a useful marker in the detection of gynecological malignancies. However, CD146 expression and detection in two other types of gynecological cancer, cervical and endometrial cancer, has not been investigated.

This study aimed to evaluate the distribution of CD146 in cervical and endometrial cancer; the diagnostic use of CD146 Abs in cervical and endometrial cancer was evaluated by calculating the correlation between clinical pathological parameters and the extent of immunohistochemical CD146 expression in tumor tissues.

## Materials and methods

### Antibody and reagents

The anti-CD146 monoclonal antibody AA4 was generated in the Protein and Peptide Pharmaceutical Laboratory (Chinese Academy of Sciences-University of Tokyo Joint Laboratory of Structural Virology and Immunology, Beijing, China). A CD31-specific antibody was purchased from Abcam (Cambridge, MA, USA). Biotin-conjugated secondary antibodies (goat anti-rabbit or -mouse) and HRP-conjugated streptavidin were purchased from Dianova (Rodeo, CA, USA). Goat anti-rabbit Alexa Fluor^®^ 488 and goat anti-mouse Alexa Fluor^®^ 555 were purchased from Invitrogen (San Diego, CA, USA). DAPI was purchased from Roche (Indianapolis, IN, USA). Normal goat serum and a 3,3′-diaminobenzidine (DAB) staining kit were purchased from ZSGB-BIO (China).

### Ethics statement

Informed consent was obtained from all participants in this study. All procedures have been approved by the Ethics Committee of Capital Medical University Affiliated Beijing Anzhen Hospital, Institute of Beijing Heart, Lung and Blood Vessel Diseases and Institute of Biophysics, Chinese Academy of Sciences.

### Clinical sampling

Malignant specimens were obtained from patients with gynecological cancer, including cervical carcinoma and endometrial carcinoma, by surgical excision, in Anzhen Hospital (Beijing, China) and the Fifth Affiliated Hospital of Zhengzhou University (Zhengzhou, Henan, China). Normal uterine cervical tissue and normal endometrium were collected by hysterectomy in non-cancer patients with multiple uterine leiomyomas or adenomyosis, respectively. None of the patients had received preoperative chemotherapy, radiotherapy or hormone therapy or suffered with malignant tumors, other than the gynecological cancer examined in this investigation.

### Immunohistochemistry

This assay was conducted according to classically established methods (14). Briefly, tissue sections were fixed with formalin and embedded with paraffin. Tissue sections were then cut (5 μm) and stained with hematoxylin and eosin (H&E) solution. Following deparaffinization, tissue sections were stained with a CD146- or CD31-specific antibody, then with biotin-conjugated secondary antibodies (1:1000), followed by HRP-conjugated streptavidin (Dianova). The sections were counterstained with hematoxylin to visualize the nuclei.

### Double-staining immunohistofluorescence analysis

Sections (5 μm) were fixed in 4% paraformaldehyde (pH 7.4) for 15 min at room temperature. Following the general procedure, which included permeabilization and blocking, the slides were co-incubated with antibodies for CD146 (mAb) and CD31 (rAb, rabbit Ab) overnight at 4°C. After washing with PBS three times, the slides were incubated with a mixture of the two secondary antibodies (anti-rabbit Alexa Fluor^®^ 488 and anti-mouse Alexa Fluor^®^ 555) in the dark for 1 h. The nuclei were stained with DAPI. Fluorescent images were acquired using a confocal microscope (FV1000, Olympus, Tokyo, Japan).

### Statistical analysis

Statistical analysis was conducted using SPSS software. The significant differences between the groups and the association between CD146 expression and different clinicopathological parameters were evaluated using the Chi-square test or Fisher’s exact test. P<0.05 was considered to indicate a statistically significant difference.

## Results

### Classification of cervical cancer specimens into categories

The collected specimens were divided into normal tissue and cancerous tissue groups. The mean age of the patients with normal and cancerous uterine cervical tissue was 44.12±10.61 and 48.25±9.86 years, respectively. The cervical samples consisted of 16 normal (control) and 256 malignant specimens (Table I). Cervical cancer is a malignant neoplasm originating from cells in the cervix uteri and consists of two histological subtypes. The majority of cervical cancer types are squamous cell carcinomas, arising in the squamous epithelial cells that line the cervix. Adenocarcinomas, arising in glandular epithelial cells, are the second most common type of cervical cancer (20). Cancer rarely occurs in other types of cells in the cervix. In this study, 241 of the cancer samples had the squamous carcinoma subtype and only 15 samples had the adenocarcinoma subtype. Cervical cancer tissue was further classified into categories following the review of existing patient medical records. With regard to the histological grade, 46 cases were grade 1 (G1), with a high differentiation phenotype, 154 tumors were moderately differentiated (grade 2, G2) and 56 tumors had a poorly differentiated phenotype (grade 3, G3). According to the International Federation of Gynecology and Obstetrics (FIGO) systems, 226 patients were in the early stages (I–II) and 30 patients were in the advanced stages (III–IV) of the disease (Table II).

### Classification of endometrial cancer specimens into categories

The mean age of the patients with normal and cancerous endometrial tissue were 33.12±10.56 and 54.69±9.43 years, respectively. Endometrial samples consisted of 18 normal (control) and 87 malignant specimens (Table I). The majority of endometrial cancer types are adenocarcinomas, originating from epithelial cells that line the endometrium and form the endometrial glands. There are numerous microscopic subtypes of endometrial carcinomas, including the common endometrioid type, the more aggressive papillary serous carcinoma and clear cell endometrial carcinomas (21). Eighty samples were endometrioid adenocarcinomas (EECs) and 7 samples were non-endometrioid adenocarcinomas (NEECs), which included 5 squamous endometrial carcinomas, 1 papillary serous endometrial carcinoma and 1 adeno-squamous endometrial carcinoma. FIGO grading showed that 30 samples were well-differentiated (G1) and 57 samples had moderate (G2) or no (G3) differentiation (Table III).

### CD146 is highly expressed in cervical and endometrial cancer

To investigate the expression of CD146 in cervical and endometrial cancer, we conducted immunohistochemical assays with AA4 mAb on all the collected specimens, which consisted of 377 samples of normal and cancerous tissue. As summarized in Table I, CD146 was detected in a large number of cancer samples. The number of true positives (Table I) in cervical and endometrial cancer was 112 and 59, respectively. The number of false negatives (Table I) in the cancerous cervical and endometrial tissue was 144 and 28, respectively. By contrast, CD146 was only detected in a limited number of normal samples. The number of false positives (Table I) in normal cervical and endometrial tissue was 0 and 7, respectively. The number of true negatives (Table I) in normal cervical and endometrial tissue was 16 and 11, respectively. Using the equation described in the legend of Table I, the positive predictive value (PPV), negative predictive value (NPV), sensitivity and specificity of CD146 expression for cervical cancer detection were calculated as 100 (112/112), 10.00 (16/160), 43.75 (112/256) and 100% (16/16), respectively. For the detection of endometrial cancer based on CD146 expression, PPV, NPV, sensitivity and specificity were 89.4 (59/66), 28.2 (11/39), 67.8 (59/87) and 61.1% (11/18), respectively.

### CD146 expression levels are positively correlated with the histological subtypes of cervical cancer

**Figure 1:**
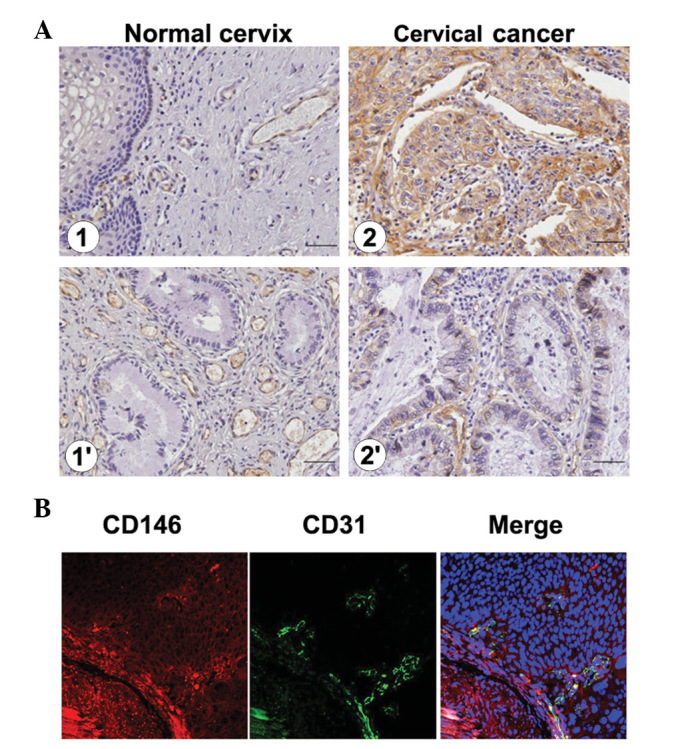
Expression of CD146 in the clinical samples of normal and cancerous cervical tissue. (A) Representative immunohistochemical staining of CD146 in vascular endothelial cells and tumor cells in the collected tissues, including: (A1) Normal cervical squamous epithelium; (A2) normal cervical mucosa glands; (A1′) cervical squamous carcinoma; and (A2′) adenocarcinoma. (B) Expression of CD146 in vascular endothelial cells was confirmed using anti-CD31 as an endothelial marker in a fluorescent immunohistochemistry assay. CD146, red; CD31, green; Merge, overlapped images of former pictures and nuclei were stained with DAPI (blue). Scale bars, 100 μm. CD146, cluster of differentiation 146.

Immunohistochemical assays showed that CD146 expression did not occur in normal cervical squamous epithelium and mucosal glands. In cervical carcinomas, CD146 was detected mainly in the membranes and cytoplasm of squamous tumor and vascular endothelial cells (Fig. 1A). This was consistent with the localization data from fluorescent immunohistochemistical assays, as shown in Fig. 1B, in which the tumor cells with CD146^+^ expression were labeled with CD146 mAb AA4 and the vascular endothelial cells with CD146^+^ expression were confirmed with an endothelial marker for CD31. The overlapping yellow regions are indicative of the co-localization of CD31 and CD146 in vascular cells. These data demonstrated that CD146 was localized in tumor cells and vascular endothelial cells in cervical carcinomas.

For patients who were <48 years old, 39% (52/133) of samples had CD146^+^ staining, whereas, for patients who were ≥48 years old, a higher number of cases were CD146^+^ and had a staining rate of 49% (60/123). There was no significant difference between the cervical cancer and normal cervical samples in the two parameters of clinical FIGO stage (I–II and III–IV stages) and histological grade (G1–G3). By contrast, as shown in Table II, the AA4^+^ reaction rate is significantly higher in squamous cervical carcinoma (112/241, 46.5%) than in cervical adenocarcinoma (0/15, 0%). This indicates that CD146 presence was a potential predictive marker (P=0.000) for discrimination of the two histological subtypes of cervical carcinoma, namely that a cervical carcinoma with higher CD146 expression levels was more likely to be the squamous carcinoma subtype than the adenocarcinoma subtype.

### CD146 expression levels are positively correlated with histological grade and the depth of the myometrial invasion in endometrial cancer

**Figure 2:**
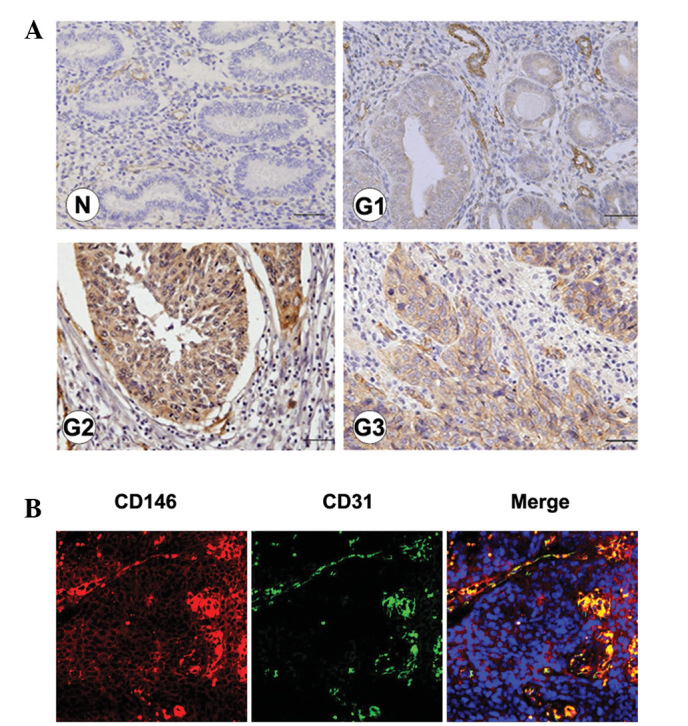
Expression of CD146 in the clinical samples of normal and cancerous endometrial tissue. (A) Representative immunohistochemical staining of CD146 in vascular endothelial cells and tumor cells in the collected tissues, including: (N) Normal hyperplasia endometrium; (G1) a highly differentiated grade of endometrial cancer; (G2) a moderately differentiated grade of endometrial cancer; and (G3) a poorly differentiated grade of endometrial cancer. (B) Expression of CD146 in vascular endothelial cells was confirmed using anti-CD31 as an endothelial marker in a fluorescent immunohistochemistry assay. CD146, red; CD31, green; Merge, overlapped images of former pictures and nuclei were stained with DAPI (blue). Scale bars, 100 μm. CD146, cluster of differentiation 146.

In normal endometrium samples, CD146 was detected in vascular endothelial cells and also in glands during the hyperplasia period. In endometrial cancer samples, CD146 was detected in the majority of tumor cells, in addition to vascular endothelial cells (Fig. 2A). The CD146 localization in vascular endothelial cells was further confirmed using fluorescent immunohistochemistry. Similar to the results in Fig. 1B, the co-localization of CD31 and CD146 in the endothelial cells of tumor blood vessels was observed in endometrial cancer (Fig. 2B).

Immunohistochemistry results showed that no statistically significant differences in CD146 expression existed between the two groups for endometrial cancer samples from patients <55 years old (70%) or ≥55 years old (65%). Furthermore, no significant difference in CD146^+^ expression was identified between cancer samples with different FIGO stages (P= 0.653). CD146^+^ staining occured in 67% (46/69) of cases in cancer samples with an early clinical stage (I–II phase), compared with 72% (13/18) in cancer samples with an advanced clinical stage (III–IV phase). For the histological subtypes of endometrial cancer, CD146 expression was detected in EEC and NEEC in 66% (53/80) and 86% (6/7) of samples, respectively, indicating that there is no significant difference in CD146 expression between the two subtypes of endometrial cancer (P=0.290).

By contrast, CD146^+^ samples showed significant differences in the histological grade and the depth of myometrial infiltration parameters. CD146*^+^* cases occured at a significantly higher rate (P= 0.036) in poorly differentiated histological grades (G2–G3) of endometrial cancer (43/57, 75%) than in the highly differentiated grade (G1) of endometrial cancer (16/30, 53%). Similarly, the depth of myometrial infiltration indicated that CD146*^+^* samples were significantly more frequent in the lesions with deep (>0.5) myometrial infiltration (32/40, 80%), compared with the lesions without or with shallow (0 or <0.5) myometrial infiltration (27/47, 57%), as evaluated by statistical analysis (P=0.025).

## Discussion

In this study, we demonstrated that the specificity, sensitivity and PPV of AA4 (a mAb for CD146) is suitable for use in the detection of cervical cancer and endometrial cancer. Results showed that CD146 expression levels were higher in cervical and endometrial cancer tissues compared with their corresponding normal tissues. Notably, CD146 expression was positively and significantly correlated with various subtypes of cervical cancer, as higher expression levels were detected in the squamous carcinoma subtype than in the adenocarcinoma subtype (Table II). The significant correlation which was identified between CD146 expression and the histological classification or the depth of myometrial invasion indicates that CD146 may be involved in the onset and development of endometrial cancer (Table III). This hypothesis was further strengthened by an immunohistofluorescent assay, where the broad expression of CD146 in the cellular membrane of malignant cancer was confirmed. Furthermore, in accordance with previous studies (22,23), immunohistofluorescence data in this study showed that CD146 was present in the majority of cancer blood vessels (Figs. 1 and 2), suggesting that CD146 may be actively implicated in the dissemination and metastasis of cervical cancer and endometrial cancer via the vascular system.

Gynecological malignant cancer, including cervical cancer, endometrial carcinoma and ovarian cancer, is life-threatening to females (24). The incidence of cervical cancer is higher than endometrial and ovarian cancer (20) and the mortality rate of ovarian cancer is the highest among these three types of cancer (25). Therefore, effective screening methods and potential therapeutic targets have been pursued in this field. At present, the clinically used biomarkers for detection of gynecological malignancies principally include squamous cell carcinoma antigen (SCC), carcinoembryonic antigen (CEA) and sugar antigens CA125, CA199 and CA153 (26–29).

However, the sensitivity and specificity are not satisfactory for the accuracy of predictive detection for gynecological malignancies (26). Therefore, seeking more reliable biomarkers is likely to aid the successful detection of tumors in the early stages of the disease and also for determining an effective therapeutic approach. Our findings of CD146 overexpression in cervical and endometrial cancer, plus the ability of AA4 to detect CD146 with high sensitivity and specificity, provides insight for further development of CD146 mAbs in the detection of malignant gynecological cancer. It also implies that a combined treatment strategy of anti-CD146 immunotherapy with other traditional chemo- or radiotherapy treatments may be a promising anticancer technique.

## Figures and Tables

**Table I:** Distribution of CD146 in normal and cancerous tissues from the cervix and endometrium.

Variable	CD146*^+^*	CD146*^−^*	Total	χ^2^	P-value	PPV	NPV	Sensitivity	Specificity
Cervical samples				11.900	<0.01	100	10.00	43.75	100
Cancer	112^a^	144^b^	256						
Normal	0^c^	16^d^	16						
Endometrial samples				5.345	<0.05	89.39	28.21	67.81	61.11
Cancer	59^a^	28^b^	87						
Normal	7^c^	11^d^	18						

CD146, cluster of differentiation 146; PPV, positive predictive value; this is the proportion of positive test results that are true positives after correction with the verdict of clinical diagnosis; PPV = true positives/(true positives+false positives) × 100 = a/(a+c) × 100. NPV, negative predictive value; this is the proportion of negative test results that are correctly diagnosed; NPV = true negatives/(false negatives+true negatives) × 100 = d/(b+d) × 100. Sensitivity, the ability to determine the individuals who have a disease; sensitivity = true positives/(true positives+false negatives) × 100 = a/(a+b) × 100. Specificity, the ability to determine the individuals who do not have a disease; specificity = true negatives/(false positives+true negatives) × 100 = d/(c+d) × 100.

**Table II:** Correlation of CD146 expression with clinicopathological parameters in cervical carcinoma.

Variable	CD146		Total	χ^2^	P-value	Correlation coefficient
	Positive, n (%)	Negative, n (%)				
Age (years)				2.435	0.119	–0.098
<48	52 (39)	81 (61)	133			
≥48	60 (49)	63 (51)	123			
Histological grade				1.830	0.401	0.080
G1	24 (52)	22 (48)	46			
G2	66 (43)	88 (57)	154			
G3	22 (39)	34 (61)	56			
FIGO stage				0.002	0.961	0.003
I–II	99 (44)	127 (56)	226			
III–IV	13 (43)	17 (57)	30			
Histological tumor type				12.393^a^	<0.001	0.220
Squamouscarcinoma	112 (46)	129 (54)	241			
Adenocarcinoma	0 (0)	15 (100)	15			

CD146, cluster of differentiation 146; G1, a highly differentiated grade of cancer, composed of glands and 5% of lesions have a solid growth pattern; G2, a moderately differentiated grade of cancer, with 6–50% of lesions composed of solid sheets of cells; G3, an undifferentiated grade, with >50% of lesions composed of solid sheets of cells. FIGO stage I–II, early stage of lesion. Stage I includes stage IA [superficial invasive cervical carcinoma (microinvasion)] and stage IB (carcinoma confined to cervix). Stage II includes stage IIA (carcinoma extends onto upper vagina) and IIB (carcinoma extends into parametrium, but does not reach pelvic side wall). Stage III includes stage IIIA (carcinoma extends onto lower vagina) and IIIB (carcinoma extends to the pelvic side wall or causes ureteric obstruction). Stage IV includes stage IVA (carcinoma involves bladder or rectum) and IVB (distant blood-borne spread).

a P<0.05 indicates a statistically significant difference.

**Table III:** Correlation of CD146 expression with clinicopathological parameters in endometrial carcinoma.

Variable	CD146		Total	χ^2^	P-value	Correlation coefficient
	Positive, n (%)	Negative, n (%)				
Age (years)				0.284	0.594	0.057
<55	31 (71)	13 (30)	44			
≥55	28 (65)	15 (35)	43			
Histological grade				4.400^a^	<0.05	–0.225
G1	16 (53)	14 (47)	30			
G2–G3	43 (75)	14 (25)	57			
FIGO stage				0.202	0.653	–0.048
I–II	46 (67)	23 (33)	69			
III–IV	13 (72)	5 (28)	18			
Depth of myometrial infiltration				5.036^a^	<0.05	–0.241
0 or <0.5	27 (57)	20 (43)	47			
>0.5	32 (80)	8 (20)	40			
Histological tumor type				1.117	0.290	–0.113
Endometrioid adenocarcinoma	53 (66)	27 (34)	80			
Non-endometrioid adenocarcinoma	6 (86)	1 (14)	7			

CD146, cluster of differentiation 146; G1, a highly differentiated grade of cancer, composed of glands and 5% of lesions have a solid growth pattern; G2, a moderately differentiated grade of cancer, with 6–50% of lesions composed of solid sheets of cells; G3, an undifferentiated grade, with >50% of lesions composed of solid sheets of cells. FIGO stage I–II, early stage of lesion. Stage I includes stage IA (tumor confined to the uterus, 0 or <0.5 myometrial invasion) and stage IB (tumor confined to the uterus, >0.5 myometrial invasion). Stage II is with cervical stromal invasion, however not beyond the uterus. Stage III includes stage IIIA (tumor invades serosa or adnexa), IIIB (vaginal and/or parametrial involvement), IIIC1 (pelvic lymph node involvement) and IIIC2 (para-aortic lymph node involvement, with or without pelvic node involvement). Stage IV includes stage IVA (tumor invasion bladder mucosa and/or bowel mucosa) and IVB (distant metastases including abdominal metastases and/or inguinal lymph nodes).

a P<0.05 indicates a statistically significant difference.

## Abbreviations:

CD146: cluster of differentiation 146
mAb: mouse antibody
CAM: cell adhesion molecule
MET-CAM: metastasis CAM
HEMCAM: hemopoietic CAM
V set: variable region
C-2 set: constant region
IgSF: immunoglobulin superfamily
PPV: positive predictive value
NPV: negative predictive value
FIGO: International Federation of Gynecology and Obstetrics systems
EECs: endometrioid adenocarcinomas
NEECs: non-endometrioid adenocarcinomas
